# Approach Motivation and Reward Sensitivity: Effects of High‐Definition Transcranial Direct Current Stimulation (HD‐tDCS) to Brain Hemispheres on Effort‐Related Cardiovascular Response

**DOI:** 10.1111/ejn.70404

**Published:** 2026-02-02

**Authors:** David Framorando, Guido H. E. Gendolla, Philip A. Gable

**Affiliations:** ^1^ Geneva Motivation Lab, FPSE, Section of Psychology University of Geneva Geneva Switzerland; ^2^ Swiss Center for Affective Sciences University of Geneva Geneva Switzerland; ^3^ Department of Psychology University of Delaware Newark Delaware USA

**Keywords:** approach/avoidance motivation, effort, pre‐ejection period, transcranial direct current stimulation (tDCS)

## Abstract

This study examined the effect of brain hemisphere stimulation on effort intensity. We applied high‐definition transcranial direct current stimulation (HD‐tDCS) to the dorsolateral prefrontal cortex (dlPFC) to manipulate left or right hemispheric activity and assess its impact on cardiovascular responses reflecting effort. In total, 102 participants (65 women, 37 men) performed a mental concentration task under right cathodal, left cathodal, or sham stimulation conditions. We recorded cardiovascular responses, including pre‐ejection period (PEP), systolic blood pressure (SBP), heart rate (HR), and diastolic blood pressure (DBP). Preregistered hypotheses predicted right cathodal stimulation to lead to greater left frontal hemispheric activity. This should result in higher effort during a mental concentration task of unclear difficulty by increasing approach motivation and thus success importance. As predicted, right cathodal stimulation increased PEP and SBP reactivity, indicating higher effort compared to the left cathodal and sham stimulation conditions. However, this effect was only evident in women, with men exhibiting a contrasting pattern. Our findings highlight the sex‐specific effects of brain stimulation on cardiovascular responses reflecting effort, with the anticipated effects appearing in women.

## Graphical abstract

Brain stimulation over the dorsolateral prefrontal cortex affects effort‐related cardiovascular responses during a mental concentration task of unclear difficulty in presence of moderate incentives. Right cathodal stimulation leads to higher physiological effort compared to left cathodal and sham stimulation. This effect is particularly pronounced in women, whereas men exhibit a contrasting response.

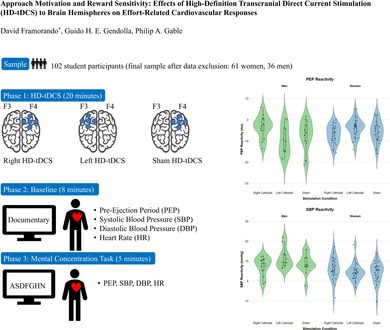

**Abbreviations**
BAS: behavioral approach system
DBP: diastolic blood pressure
EEG: electroencephalography
FHA: frontal hemispheric activity
HR: heart rate
LCS: left cathodal stimulation
PEP: pre‐ejection period
RCS: right cathodal stimulation
RST: reinforcement sensitivity theory
SBP: systolic blood pressure
SR: sensitivity to rewards
tDCS: transcranial direct current stimulation
TMS: transcranial magnetic stimulation

## 1 Introduction

Our behavior is not influenced by a single static force, but by the dynamic interplay between two fundamental and opposing motivational systems. Approach motivation encourages an organism to move toward an object. By contrast, avoidance motivation is the tendency or appeal of an organism to move away from or withdraw from an object (Harmon‐Jones et al. 2013; Lacey and Gable 2021). While previous research has nearly exclusively used behavioral manipulations or self‐report measures of approach and avoidance motivation (e.g., Heponiemi et al. 2003; Peterson et al. 2008; Schiff and Lamon 1989; see Harmon‐Jones and Gable 2018; Kelley et al. 2017 for recent reviews), we took a neuromodulatory approach. Our present study tested if a neural manipulation of approach motivation via high‐definition transcranial direct current stimulation (HD‐tDCS) of the brain has effects on cardiovascular responses reflecting the intensity of effort during the performance of a cognitive task.

### 1.1 Approach Motivation, Sensitivity to Rewards (SR), and Effort

According to reinforcement sensitivity theory (RST; Gray and McNaughton 2000; McNaughton 2025), approach motivation is grounded in a behavioral approach system (BAS) that responds to reward and non‐punishment signals (Gray 1970): Increased BAS activation is associated with increased SR. Importantly, SR plays a crucial role in determining effort—the mobilization of resources for action execution (Gendolla and Wright 2009).

According to motivational intensity theory (Brehm and Self 1989), rewards determine success importance: The higher the positive consequence to be obtained, the higher is success importance. In motivational intensity theory, success importance is expected to directly determine the intensity of effort when task difficulty is unspecified because it is unclear or unfixed (i.e., when the performance standard is unknown or when the performance standard can be chosen by the individual; Brehm and Self 1989; Richter 2012; Wright 1996). Consequently, tasks of unclear or unfixed difficulty allow us to test the direct impact of rewards on resource mobilization in goal pursuit, providing a strong rationale for employing such tasks to test our predictions.

In tasks of unspecified difficulty, individuals with stronger SR are expected to assign greater value to the positive consequences of reward. If receiving a reward is contingent on successful completion of a task, then these individuals should be more motivated to succeed (higher success importance) than those with lower SR (e.g., Brinkmann et al. 2014; Franzen and Brinkmann 2015; Franzen et al. 2019). Overall, this means that changes in SR should affect effort in the presence of rewards; the higher the SR, the stronger the effect of rewards on success importance and the higher the effort when task difficulty is unspecified. This is supported by research on anhedonia, where decreased SR has been shown to lead to lower success importance and thus lower effort when task difficulty is unspecified (e.g., Franzen and Brinkmann 2015).

Given the established relationship between approach motivation and heightened SR, and the impact of SR on effort in tasks of unspecified difficulty, we hypothesize that enhancing approach motivation may increase effort in reward‐based tasks of unclear difficulty. Prior studies have usually assessed individual differences in approach motivation with self‐report measures or have measured neural correlates of approach motivation using EEG to assess changes in hemispheric asymmetry. Only a few studies have induced asymmetries through behavioral manipulations—such as unilateral muscle contractions (hand or facial) or nostril breathing (see Harmon‐Jones and Gable 2018; Kelley et al. 2017, for reviews). However, with these manipulations, it is difficult to determine which cortical areas are being manipulated, or whether shifts in neural activity could be correlates of the behavioral change. There is a lack of empirical research that directly manipulates approach motivation on the neural level to test its causal effect on effort. Brain stimulation techniques, which can modulate neural activity associated with motivational systems, offer a promising avenue for this manipulation (see Gable et al. 2018; Harmon‐Jones and Gable 2018; Kelley et al. 2017 for reviews). Therefore, we have manipulated approach motivation through brain stimulation and assessed its effect on cardiovascular responses reflecting effort in a task of unclear difficulty.

### 1.2 Frontal Hemispheric Asymmetries

Neurobiological research has established a link between approach motivation and relative differences in activity between the left and right frontal hemispheres, commonly referred to as frontal hemispheric activity (FHA). Electroencephalography (EEG) studies have demonstrated that greater activity in the left frontal hemisphere relative to the right (greater relative left FHA) is associated with approach motivation assessed with the BIS/BAS questionnaire (Coan and Allen 2003; Dawson et al. 1992; Harmon‐Jones and Allen 1997, 1998; Harmon‐Jones et al. 2009; Sutton and Davidson 1997). Importantly, researchers have attempted to induce approach motivation by modifying frontal hemispheric asymmetries using transcranial direct current stimulation (tDCS) and transcranial magnetic stimulation (TMS) to manipulate cerebral activation (Fecteau et al. 2007; Ohmann et al. 2018; Knoch, Gianotti, et al. 2006; see Harmon‐Jones and Gable 2018; Kelley et al. 2017, for reviews). TMS uses magnetic pulses to modulate brain activity and shift contralaterally, for example, by applying them to the right prefrontal cortex to increase approach‐oriented behavior by enhancing greater relative left FHA (Knoch, Gianotti, et al. 2006; Knoch, Pascual‐Leone, et al. 2006). In contrast, tDCS uses weak electrical current between two electrodes to alter cortical excitability, with anodal stimulation increasing and cathodal stimulation decreasing excitability (Nitsche and Paulus 2000). Configurations such as left anodal/right cathodal (LAS/RCS) can increase greater relative left FHA for approach motivation (Dambacher et al. 2015; Hortensius et al. 2012).

Recent technological advancements have enabled the focal targeting of brain areas using HD‐tDCS. Unlike conventional tDCS, which uses two larger pad‐electrodes, HD‐tDCS employs multiple smaller electrodes in a 4 × 1 ring configuration, with a central electrode surrounded by four return electrodes (Kuo et al. 2013). This setup isolates the stimulated region, allowing for more precise brain stimulation with longer‐lasting effects (Kuo et al. 2013; Parlikar et al. 2021). Accordingly, HD‐tDCS may be more effective in modulating FHA compared to conventional tDCS, with more robust and consistent effects reported for both anodal and cathodal stimulation (e.g., Sehatpour et al. 2021; see Parlikar et al. 2021 for recent review). On this basis, we sought to examine FHA‐related changes on effort‐related cardiovascular responses by applying inhibitory cathodal HD‐tDCS to the left or right dorsolateral prefrontal cortex (dlPFC) (Mosayebi‐Samani et al. 2023; Sehatpour et al. 2021; Smirni et al. 2015) to test our hypothesis concerning the role of approach motivation on effort intensity.

### 1.3 Frontal Hemispheric Asymmetries, Sensitivity to Rewards, and Mental Effort

As outlined above, greater relative left FHA is reliably associated with approach motivation and increased responsiveness to rewards (Coan and Allen 2003; Harmon‐Jones and Gable 2018; Kelley et al. 2017). Because reward sensitivity determines the perceived importance of success—a central determinant of effort according to MIT in tasks of unspecified difficulty (Brehm and Self 1989)—greater relative left FHA should lead to higher resource mobilization when rewards are present. Importantly, although FHA has been extensively studied as a correlate of approach motivation, its effect on effort intensity has not yet been directly tested. The present study addresses this gap by manipulating relative FHA through HD‐tDCS applied to the dlPFC and assessing its impact on effort‐related cardiovascular responses during a task of unclear difficulty.

### 1.4 Measuring Effort

Most research on effort intensity within MIT (Brehm and Self 1989) has used indicators of sympathetic nervous system impact on the heart to test the theory's predictions (see Gendolla et al. 2012, 2019; Richter et al. 2016, Wright and Kirby 2001 for overviews). The underlying rationale draws on seminal work by Wright (1996), who built on Obrist's (1981) findings of beta‐adrenergic sympathetic impact of the cardiovascular system when individuals engage in tasks where performance determines outcomes (active coping tasks). Wright suggested that sympathetic myocardial activity in such tasks reflects effort intensity. Importantly, sympathetic myocardial activity is most reliably indexed by the cardiac pre‐ejection period (PEP)—the interval between ventricular depolarization and the opening of the aortic valve (Berntson et al. 2004)—as measured by impedance cardiography (ICG). The PEP is highly sensitive to reward manipulations when task difficulty is unclear (Richter and Gendolla 2009). As cardiac contractility also affects cardiac output (the volume of blood pumped by the ventricles per minute), earlier studies have used systolic blood pressure (SBP) to measure effort (Gendolla et al. 2012; Richter et al. 2016; Wright and Kirby 2001 for reviews). However, SBP is also influenced by peripheral vascular resistance, which is not systematically influenced by beta‐adrenergic activity (Levick 2003). Other studies have relied on heart rate (HR) to monitor effort (e.g., Rogers 1969). However, HR is also influenced by the parasympathetic nervous system. Therefore, changes in PEP during task performance are the most sensitive and reliable effort index among these measures (Kelsey 2012), as also illustrated by past work (e.g., Chatelain and Gendolla 2015; Framorando and Gendolla 2018, 2019a, 2023; Framorando et al. 2024a; Lasauskaite Schüpbach et al. 2014; Freydefont et al. 2012). Accordingly, PEP reactivity during task performance is a reliable marker of effort intensity (Albinet et al. 2024; Gendolla 2025a).

Importantly, PEP should always be measured along with HR and DBP to, respectively, monitor possible preload (ventricular filling) or afterload (arterial pressure) effects on PEP. PEP responses should only be attributed to beta‐adrenergic sympathetic activity when decreases in PEP occur without simultaneous decreases in HR or blood pressure (Sherwood et al. 1990).

### 1.5 The Present Experiment

We investigated the effect of applying HD‐tDCS to dlPFC on effort‐related cardiovascular responses during a mental concentration task. Participants engaged in a task of unclear difficulty (i.e., without any information about success criteria, number of trials, or task duration) and could earn money for their success. Following the neuromodulatory principles observed with TMS, in which inhibition of the right hemisphere increases activity in the left hemisphere, relative FHA was induced using left versus right cathodal HD‐tDCS (e.g., Fecteau et al. 2007; Smirni et al. 2015; see Schutter et al. 2004 for a review). In addition, a right sham stimulation was added as a control.

**FIGURE 1:**
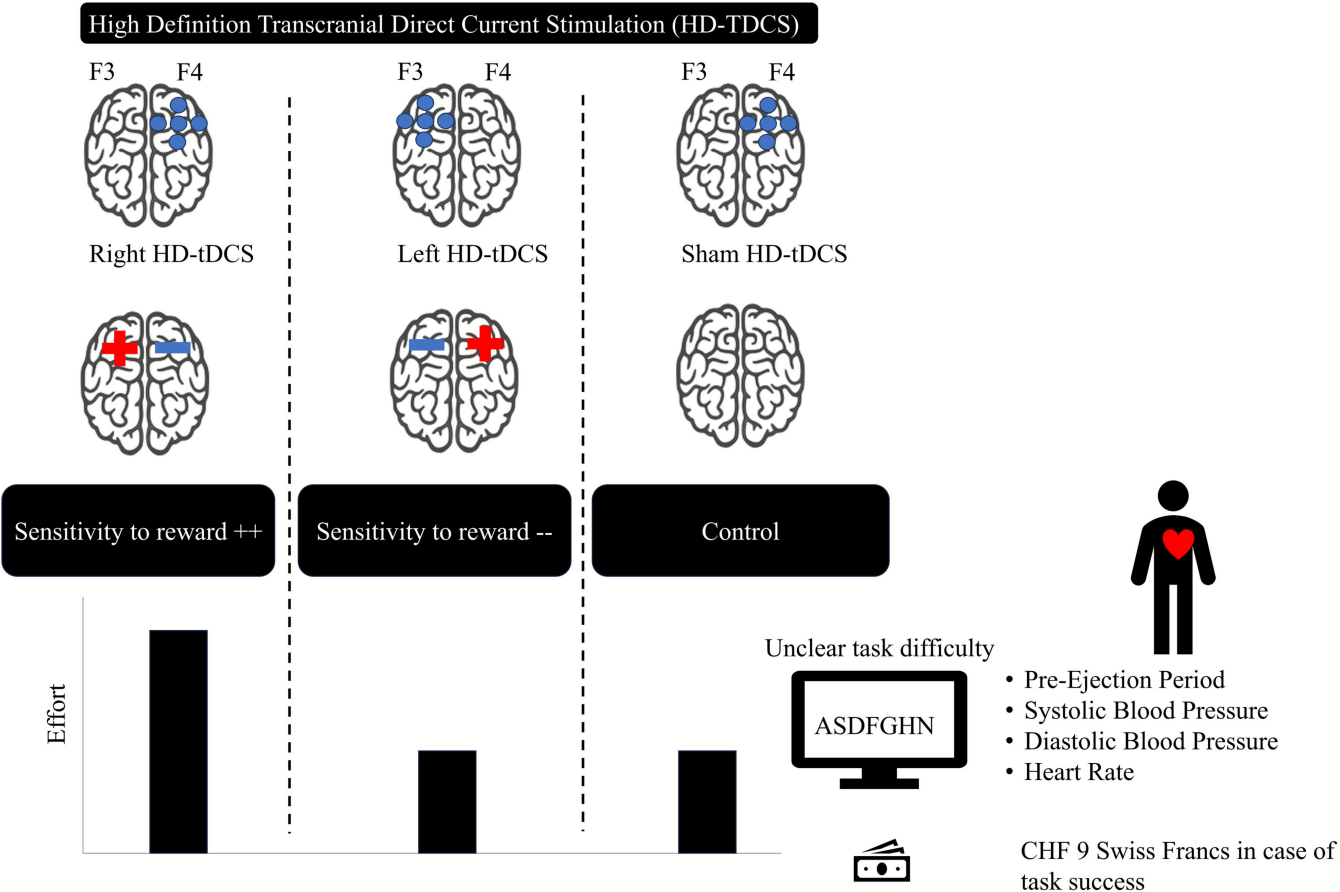
Hypothesized effects of stimulation‐induced frontal hemispheric asymmetry on approach motivation, sensitivity to reward, and effort.

As illustrated in Figure 1, we hypothesized that RCS should shift hemispheric activity, leading to greater left FHA, and thus to increased approach motivation and SR, which, combined with monetary incentive, should cause higher effort in an unclear difficulty task. In contrast, we anticipated that LCS (resulting in greater relative right FHA) and the sham condition (which would not alter the activity balance between the hemispheres) would lead to moderate SR and lower effort in the presence of incentives during unclear task demands.

## 2 Materials and Methods

### 2.1 Participants and Design

The analysis plan for this study was preregistered on AsPredicted (https://aspredicted.org/2M3_RVF). We recruited 102 student participants from various Faculties at the University of Geneva, aiming to ensure at least 30 valid datasets per group. This sample size was determined to align with a previous study on brain stimulation and cardiovascular measures of effort (Framorando et al. 2021) and was deemed sufficient to detect a Bayes Factor greater than 10, indicating strong evidence for our hypotheses (Van Doorn et al. 2021). Participants received CHF 30 (approximately EUR 32.18) as compensation for their time and were randomly assigned to one of the experimental conditions in a three‐group between‐persons design (right cathodal, left cathodal, or sham stimulation). After an initial check of the signal quality and outliers, five participants were excluded from the main analysis: one due to electrocardiogram (ECG) or ICG signal loss, two for extremely low response accuracy in the cognitive task (< 3 SDs from the condition mean), and two due to excessive SBP and/or HR reactivity.^1^ This resulted in a final sample of *N* = 97 (61 women and 36 men) with a mean age of 23.73 years (SE = 0.43; Median = 22.00; Range = 18–39).^2^

### 2.2 Physiological Measures

We used a Cardioscreen 1000 system (Medis, Ilmenau, Germany) to measure PEP and HR based on ECG and ICG signals. Four pairs of single‐use electrodes (Ag/AgCl; Medis, Ilmenau, Germany) were placed on the left and right sides of the participants' neck and chest (left middle axillary line at the height of the xiphoid). The signals were amplified, converted to digital data (sampling rate 1000 Hz), and analyzed offline (50‐Hz low‐pass filter) with BlueBox 2. V1.22 software (Richter 2010). The R‐peaks were automatically identified using a threshold peak detection algorithm and visually confirmed, allowing HR determination. The first derivative of the change in the thoracic impedance was calculated, and the resulting dZ/dt signal was averaged over a 1‐min period based on the detected R‐peaks. The location of point B was estimated based on the RZ interval of the valid cardiac cycles (Lozano et al. 2007). The identified point B locations were visually inspected and manually corrected, if necessary, following the recommendations by Sherwood et al. (1990). This latter step was made on the raw data level by one of the authors, who was unaware of the experimental condition of the participants and the condition *Ms* during this process. PEP (ms) was defined as the interval between the onset of R in the ECG signal and point B in the ICG signal (Berntson et al. 2004). HR was determined based on ECG interbeat intervals assessed using the Cardioscreen system.

SBP and DBP were measured oscillometrically at 1‐min intervals using a Dinamap ProCare monitor (GE Healthcare, Milwaukee, WI, USA). The blood pressure cuff, which inflated automatically at 1‐min intervals, was placed over the brachial artery above the elbow of the nondominant arm.

For researchers interested in more detailed hemodynamic responses that were unrelated to our hypotheses, analyses of cardiac output and total peripheral resistance are accessible in the online Supporting Information.

### 2.3 Application of tDCS

Brain stimulation was provided using a Neuroelectric StarStim 8‐channel device (Barcelona, Spain). The locations of the target electrodes were determined using a 10–20 EEG system (Jasper 1958). The cathode was placed over F3 to induce LCS and the anodes over AF3, F1, F5, and FC3. To induce RCS, the cathode was placed over F4, whereas the anodes were placed over AF4, F2, F6, and FC4. tDCS was applied for 21 min at an intensity of 2 mA, including 30‐s ramp‐on and 30‐s ramp times for the current. In addition, in the sham (control) condition, electrodes were placed at the same position as in the RCS condition, but the stimulator was turned off after 30 s. Participants in the sham condition received the same instructions as those in the LCS and RCS conditions.

To ensure blinding, an external technician prepared the stimulation files, which were labeled only with participant number, sex (M = Male, F = Female), and hemisphere. For participants assigned to right hemisphere stimulation, only the technician knew whether the file corresponded to active or sham stimulation. The tDCS system automatically concealed the stimulation type during administration, ensuring that both the experimenter and the authors remained unaware of whether a participant received active or sham stimulation. This double‐blind configuration makes expectancy effects highly unlikely.

### 2.4 Procedure

All procedures and measurements were approved by the ethics commission of the State of Geneva (Project‐ID: 2023‐01563), and all participants gave informed consent before the procedure started. To avoid experimenter effects (e.g., Gilder and Heerey 2018), the experimenter was hired and unaware of both the hypotheses and experimental conditions (see Section 2.3). When subscribing to the experiment, participants were asked not to consume caffeinated beverages (e.g., tea, coffee, or cola) or to exercise for at least 2 h before their session appointment. Before the experimental sessions, participants completed a safety questionnaire indicating whether they suffered from epilepsy, had a pacemaker or other medical implants, a history of brain injury, or did currently use psychoactive drugs (e.g., cannabis and cocaine).^3^ They also needed to read and sign a written informed consent form outlining the potential effects of tDCS and to complete the BIS/BAS scale (Carver and White 1994). For safety reasons, individuals with epilepsy, headaches, or metallic implants in the head were excluded. Additionally, all the participants read and signed a consent form detailing the experimental procedures. On arrival, they were welcomed, seated in a comfortable chair in front of a computer. Before starting the experiment, the participants were equipped with the tDCS and physiological sensors. The experimenter then started the computer program using an experimental protocol (E‐Prime 3.0; Psychology Software Tools, Pittsburgh, PA, USA).

The protocol started with biographical questions (age and sex)^4^ and a baseline affective state measure with items of the UWIST Mood Adjective Checklist (Matthews et al. 1990) and additional items (two sadness items: sad and down; two anger items: angry and irritating; two happiness items: happy and joyful; two fear items: frightened and anxious) on 7‐point scales (1 = *not at all*, 7 = *very much*). The affect ratings were introduced as standard measures to consider potentially different feeling states of the participants. Next, the participants watched a hedonically neutral documentary about trees (20 min), while tDCS was administered to minimize their focus on the sensation of stimulation. This was followed by another neutral documentary about space (8 min), during which cardiovascular baseline activity was recorded. The participants were instructed to remain passive and relaxed. After the baseline period, participants learned that they would perform a Sternberg‐type short‐term memory task. Before starting the task, participants were informed they would earn an additional 9 CHF (approximately EUR 9.60) upon successfully completing the task. However, to keep the task difficulty unclear, they were not given the success criteria. Also, participants did not complete any practice trials for the same reason.

**FIGURE 2:**
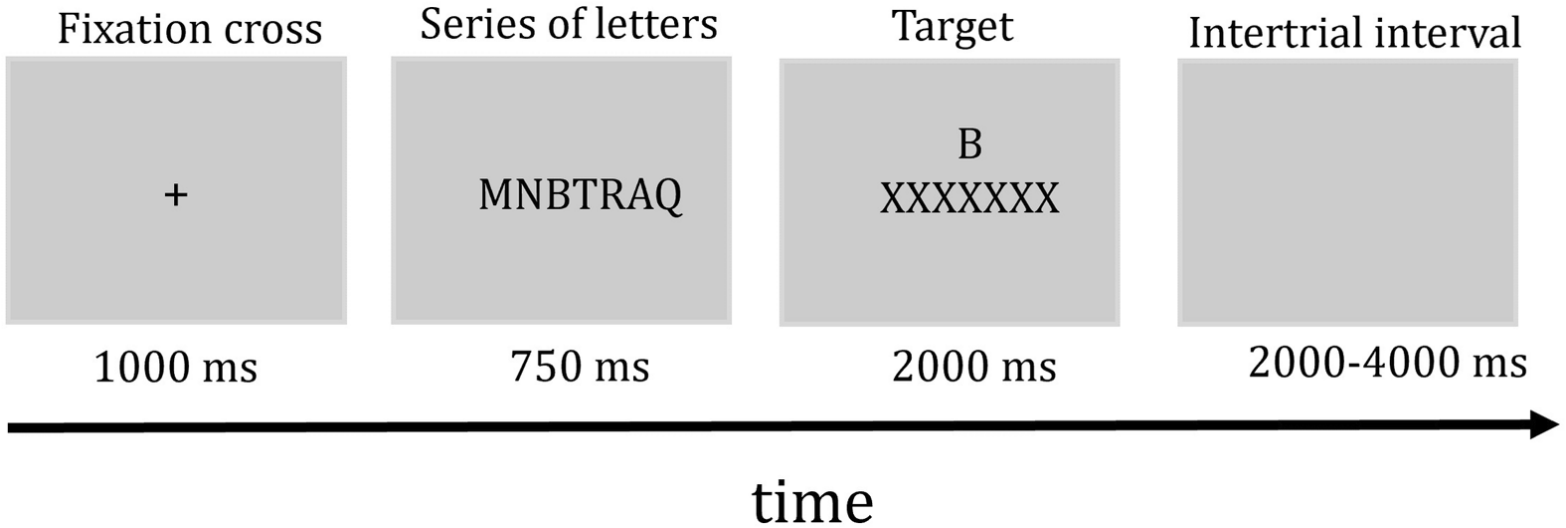
Example of an experimental trial. *Note:* In the example, the letter series “MNBTRAQ” is displayed, which includes the target letter “B.” Participants should respond “yes” by pressing the green keyboard key.

As depicted in Figure 2, trials started with a fixation cross presented for 1000 ms. Then a string of 3 to 12 letters appeared for 750 ms, followed by a backward mask consisting of a row of the letter “X.” A target letter appeared above the mask, and participants were asked to indicate within 2 s whether that letter was part of the previously shown string by pressing “yes” (green keyboard key) or “no” (red keyboard key). Importantly, participants had no information regarding the number of trials, the duration of the task, the length of the letter series, or any success criteria rendering the task's difficulty unclear.

The task consisted of 35 experimental trials without any performance feedback. After each response, the note “response entered” was presented for a duration of 4 s minus the participant's previously assessed reaction time in that trial. If there was no response within 2 s, participants received the message “Please respond faster.” The intertrial interval was randomly selected from 2 to 4 s.

After the task, the participants rated the same eight affect items as those at the beginning of the procedure. Finally, they answered additional questions regarding their native language, French language skills, cardiovascular health status, and eventual medication. Participants who did not understand French or were taking medication—particularly beta‐blockers—were excluded from the sample. The experiment finished with a debriefing, in which participants were informed about their scores and whether they succeeded in the task.

### 2.5 Data Analysis

The data and data coding are available on Yareta, the open‐access data archiving server at the University of Geneva: https://doi.org/10.26037/yareta:xrpzpx6psng3znp5f6e6lpem7e. The data analysis process was preregistered on the Aspredicted platform (https://aspredicted.org/2M3_RVF).^5^ Analyses were performed using R studio (R Core Team 2023) and JASP (JASP Team 2023) software.

To test the effect of HD‐tDCS on cardiovascular responses, we employed Bayesian analyses using Bayes factors to compare our hypothesized pattern of effects against the null hypothesis (Masson 2011). The Bayesian approach was chosen because it allows for the quantification of evidence in favor of both the null and alternative hypotheses, providing a more nuanced interpretation of the data compared to traditional null‐hypothesis significance testing (Dienes 2014; Rouder et al. 2009). We hypothesized that right cathodal stimulation (RCS) would be associated with higher effort‐related cardiovascular reactivity compared to left cathodal stimulation (LCS) and the sham condition. To evaluate this prediction, we modeled an a priori contrast with weights of −2 for the RCS condition and +1 for both the LCS and sham conditions (see preregistered report: https://aspredicted.org/2M3_RVF).

## 3 Results

### 3.1 BIS/BAS Scores

To check for potential dispositional differences in approach/avoidance motivation, preliminary one‐way Bayesian ANOVAs were conducted with condition as the factor for BIS, reward responsiveness, drive, and fun‐seeking scores. The resulting Bayes factors and posterior probability were low for reward responsiveness, drive, and fun‐seeking (BFs ≤ 0.352, *P(M∣data)* < 0.260). For BIS, the Bayes Factor (BF = 2.428) and the posterior probability, *P(M∣data)* = 0.708, suggest only weak to moderate evidence for a difference across conditions. These findings suggest limited support for condition‐based differences in BIS/BAS scores at baseline.

### 3.2 Cardiovascular Baselines

Following previous studies (e.g., Framorando et al. 2023, 2024a, 2024b), we had a priori decided to calculate participants' cardiovascular baseline values by averaging the measures taken during the last 3 min of the habituation phase. This was done because cardiovascular activity typically stabilizes after some minutes of rest. These scores showed high internal consistency (Cronbach's *α*s ≥ 0.95). Preliminary one‐way Bayesian ANOVAs were conducted with condition as the factor for each baseline score. The resulting Bayes factor and posterior probability were low (BFs < 0.605, *P(M∣data)* < 0.377). These findings suggest limited support for condition‐based differences at baseline. Baseline cell means are presented in Table 1.

**TABLE 1:** Cell means and standard errors (in parentheses) of cardiovascular baseline scores.

	Men			Women		
	Right cathodal	Left cathodal	Sham	Right cathodal	Left cathodal	Sham
PEP	105.10 (2.61)	104.15 (2.59)	99.89 (3.69)	104.51 (1.96)	97.94 (2.15)	100.60 (2.16)
SBP	102.67 (2.35)	111.17 (3.29)	102.97 (2.42)	99.92 (1.41)	97.06 (1.44)	95.32 (1.41)
HR	76.13 (2.98)	73.91 (3.09)	68.53 (3.21)	76.53 (2.82)	74.14 (2.79)	78.07 (2.63)
DBP	59.90 (1.73)	58.79 (1.42)	59.07 (1.52)	60.43 (1.24)	58.08 (1.17)	56.67 (1.56)

Abbreviations: DBP = diastolic blood pressure (in mmHg), HR = heart rate (in beats/min), PEP = pre‐ejection period (in ms), SBP = systolic blood pressure (in mmHg).

### 3.3 Cardiovascular Reactivity

We calculated cardiovascular reactivity scores by subtracting the participants' baseline scores from the average values of the five 1‐min values of PEP, SBP, HR, and DBP assessed during task performance. These scores showed high internal consistency (Cronbach's *α* ≥ 0.80). To test for potential moderation of the expected effect over time, we conducted a 3 (stimulation condition: right cathodal, left cathodal, and right sham) × 5 (time: task minutes) mixed‐model Bayesian ANOVA for each physiological measure. The analysis revealed weak evidence for an interaction effect between stimulation condition and time. For PEP, SBP, and HR reactivity, the best‐fitting model included only the main effect of time (BF > 672.231, *P(M|data)* > 0.884), while the model testing the stimulation condition × time interaction showed low Bayes Factor and posterior probability (BF < 0.028, *P(M|data)* < 0.027). For DBP, both the stimulation condition and time factors demonstrated a low model fit (BFs < 0.089, *P(M|data)* < 0.072). Given the absence of support for moderations of the stimulation effect by stimulation condition contrast × time interactions, our subsequent analyses were applied on the *M*s of the five 1‐min reactivity scores of PEP, HR, SBP, and DBP for task performance.

We also examined whether the expected effect of brain stimulation on cardiovascular reactivity was moderated by sex. Prior work suggests sex differences in tDCS effects—especially over the dlPFC—with more pronounced effects in women (e.g., Gao et al. 2018; Lapenta et al. 2012; Palmisano et al. 2021; Weller et al. 2023; Yang et al. 2018). Accordingly, we tested a 3 (stimulation condition: right cathodal, left cathodal, and right sham) × 2 (sex: women and men) interaction on PEP, our main effort‐related cardiac index, which revealed strong support for the model (BF = 10.081, *P(M|data)* = 0.91). Consequently, the a priori condition contrasts were run separately for women and men.

#### 3.3.1 PEP Reactivity^6^

**FIGURE 3:**
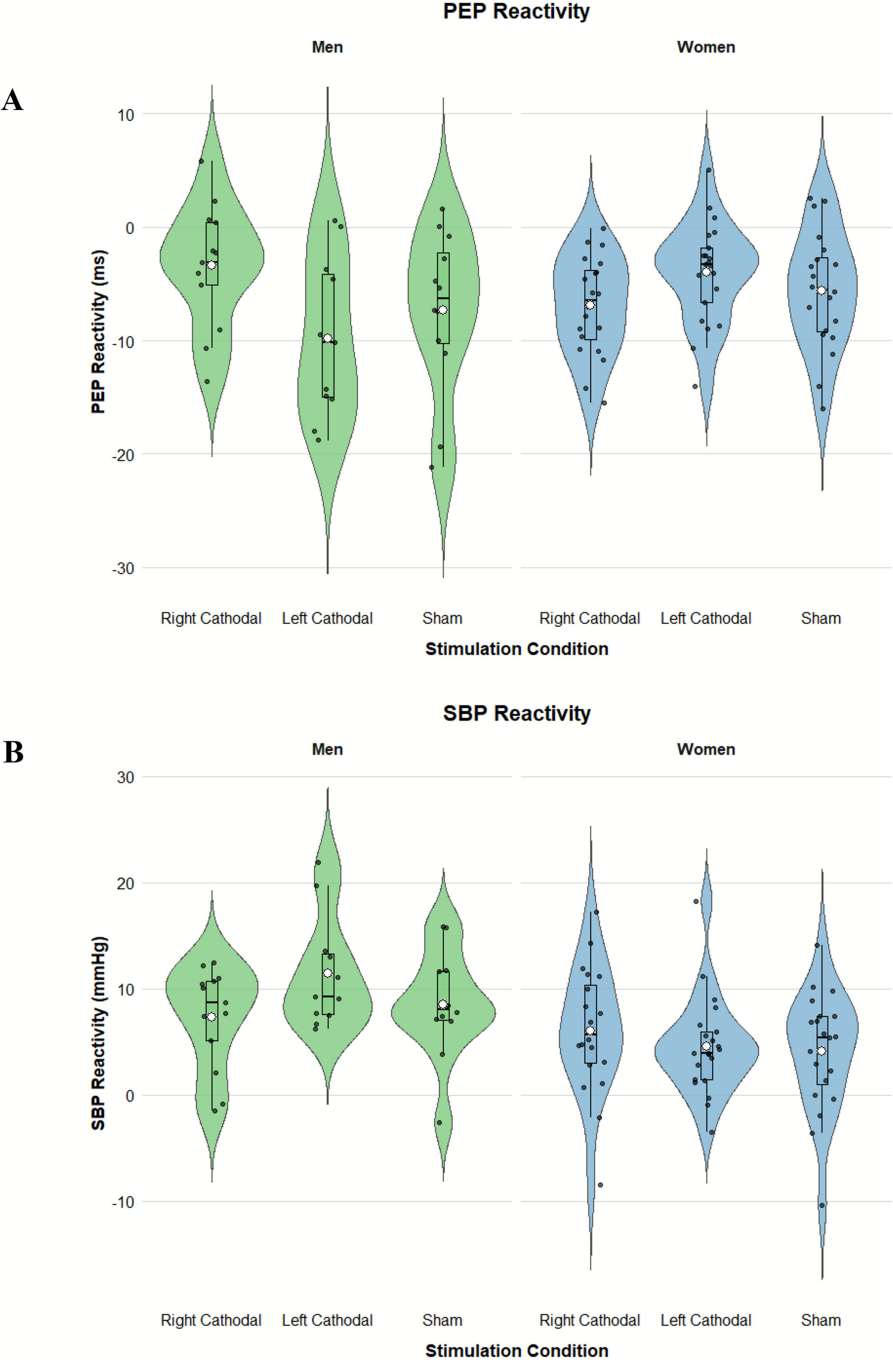
Distribution of pre‐ejection period (PEP (in ms) and systolic blood pressure (SBP) (in mmHg) reactivity across experimental conditions, separately for men and women. Violin plots depict the full distribution of individual reactivity scores within each condition.

The Bayes factors indicated strong evidence supporting the model (stimulation condition: right cathodal ‐2, left cathodal +1, and sham +1) for women (BF = 11.882, *P(M|data)* = 0.924), but weak evidence for men (BF = 0.033, *P(M|data)* = 0.031). For women, PEP reactivity corresponded to our effort‐related hypothesis. As expected, and illustrated in Figure 3, PEP reactivity for women was stronger following RCS (M = −6.93, SE = 0.97) compared to left cathodal (M = −3.98, SE = 0.97) and right sham (M = −5.61, SE = 1.15) stimulation conditions.^7^

#### 3.3.2 SBP Reactivity

The Bayes Factor provided moderate evidence supporting the model (stimulation condition: right cathodal +2, left cathodal −1, and sham −1) for women (BF = 4.937, *P(M|data)* > 0.828), but weak evidence for men (BF = 0.120, *P(M|data)* = 0.107). As expected, and shown in Figure 3, SBP reactivity was stronger after RCS (M = 6.05, SE = 1.32) compared to left cathodal (M = 4.57, SE = 1.01) and right sham (M = 4.11, SE = 1.25) stimulations.^8^

#### 3.3.3 HR and DBP Reactivity

The a priori contrasts for men and women did not reveal strong or moderate evidence supporting the model (BFs < 1.02, *P(M|data)* > 0.506). Cardiovascular reactivity *Ms* and *SEs* appear in Table 2.

**TABLE 2:** Cell means and standard errors (in parentheses) of cardiovascular reactivity scores.

	Men			Women		
	Right cathodal	Left cathodal	Sham	Right cathodal	Left cathodal	Sham
HR	3.98 (1.14)	3.82 (1.10)	4.14 (0.88)	2.71 (1.12)	2.71 (0.73)	3.08 (1.39)
DBP	4.75 (1.13)	5.41 (0.74)	4.68 (1.03)	2.52 (0.83)	2.78 (0.69)	3.20 (0.93)

Abbreviations: DBP = diastolic blood pressure (in mmHg), HR = heart rate (in beats/min).

### 3.4 Task Performance

Bayesian ANOVAs for response accuracy (percentage of correct responses across the 35 trials) and reaction times (mean reaction times for correct responses across the 35 trials) showed that the results mostly supported the null effect models (response accuracy: *P(M|data)* = 0.655; reaction times: *P(M|data)* = 0.728). Models including stimulation condition, sex, and stimulation condition × sex interaction effects revealed lower posterior distribution fits (response accuracy: BFs < 0.251, *P(M|data)* < 0.165; reaction times: BFs < 0.221, *P(M|data)* < 0.161). This provides little evidence that the stimulation condition, sex, or their interaction affected task performance. Participants exhibited a mean response accuracy of 80.17% (SE = 0.77) and a mean reaction time of 967.11 milliseconds (SE = 13.60).

### 3.5 Verbal Measures

#### 3.5.1 Experienced Affect

Following previous studies (e.g., Framorando et al. 2023, 2024a, 2024b), we calculated sum scores for sadness, happiness, anxiety, and anger for the pre‐task and post‐task affect measures. These sum scores were subjected to Bayesian 3 (stimulation condition) × 2 (sex) × 2 (time) mixed‐model ANOVAs.

The Bayesian ANOVAs indicated that the best‐fitting model for both the happiness and anxiety scores included only the time factor (happiness: BF = 82.312, *P(M|data)* = 0.533; anxiety: BF = 35.103, *P(M|data)* = 0.180). Models including the stimulation condition, sex, time, or their interactions revealed lower posterior distribution fits (happiness: BF = 0.335, *P(M|data)* = 0.181; anxiety: *BF* = 0.977, *P(M|data)* = 0.176). Happiness and anxiety scores were higher before (happiness: M = 8.61, SE = 0.27; anxiety: M = 4.57, SE = 0.24) than after the task (happiness: M = 7.77, SE = 0.30; anxiety: M = 3.71, SE = 0.20).

For sadness and anger, the null models were the most supported (sadness: *P(M|data)* = 0.553; anger: *P(M|data)* = 0.282). Models including the stimulation condition, sex, time, or their interactions found lower posterior distribution fits (sadness: BF < 0.259, *P(M|data)* < 0.144; anger: BF < 0.879, *P(M|data)* < 0.247). Taken together, this suggests that the tDCS did not induce negative or positive feelings, as there was no evidence for changes due to the stimulation conditions or their interactions with sex over time.

#### 3.5.2 Funnel Debriefing

No participant complained about itching or pain related to the experimental tDCS manipulation in any stimulation condition.

## 4 Discussion

This study is the first to demonstrate the effects of HD‐tDCS on effort‐related cardiovascular responses in a cognitive task with monetary incentive. As predicted, Bayesian statistical analysis supported the model showing that participants—especially women—exhibited increased effort during task performance after RCS than after LCS and sham stimulations. The analysis revealed strong evidence of changes in PEP reactivity, our primary effort‐related measure, and changes in SBP reactivity. Importantly, post hoc Bayesian statistical analysis revealed the opposite pattern for men with stronger effort‐related cardiovascular responses after LCS than after RCS and sham conditions. These findings suggest that different brain stimulation conditions affect effort intensity differently based on sex.

### 4.1 Frontal Hemispheric Asymmetry, Approach Motivation, and Effort

The pattern observed in the female sample is compatible with the idea that RCS has increased relative left FHA, which increases approach motivation and leads to heightened SR (e.g., Coan and Allen 2003; see Harmon‐Jones and Gable 2018; Kelley et al. 2017 for reviews). This elevated SR likely caused individuals to place greater importance on achieving success when incentives were present. In situations where task difficulty is unclear, MIT posits that the importance of success becomes the primary determinant of effort (Brehm and Self 1989; see Gendolla et al. 2012, 2019; Richter et al. 2016 for recent reviews). Consequently, the enhanced SR resulting from increased approach motivation in the context of small monetary incentives likely led to greater effort due to the high success importance during an unclear task demand. Importantly, our study is the first to demonstrate a link between approach motivation and MIT, showing that HD‐tDCS‐induced changes in relative FHA can influence effort‐related cardiovascular responses in the presence of monetary incentives.

### 4.2 Sex Differences in Neuromodulation Effects

While the sex effects in our study were not expected a priori, recent studies have identified a growing body of evidence that the effects of tDCS, particularly over dlPFC, may vary significantly between sexes (e.g., Gao et al. 2018; Lapenta et al. 2012; Palmisano et al. 2021; Weller et al. 2023; Yang et al. 2018). For instance, Gao et al. (2018) found that men and women responded differently to tDCS over the dlPFC during a “cheap talk” sender–receiver game. In this game, the sender holds private information that the receiver lacks and can choose to communicate truthfully or deceptively to maximize personal gain, as there is no direct penalty for lying. Gao et al. (2018) found that right anodal/left cathodal tDCS to the dlPFC reduced deceptive behavior in women but had no significant effect on men. Similarly, Weller et al. (2023) found sex differences in the effects of tDCS on cognitive training, with women showing greater performance gains than men in a challenging arithmetic task when tDCS was applied during the task. Martin et al. (2017) also reported that participants' sex mediates the effects of HD‐tDCS on the performance in the Reading the Mind in the Eyes Test (Baron‐Cohen et al. 2001), showing improved outcomes for women after anodal stimulation to the dorsomedial prefrontal cortex (dmPFC). These findings collectively suggest that women may exhibit greater sensitivity to neuromodulation techniques like tDCS, which could explain the sex difference found in our present experiment.

Several neurobiological mechanisms have been proposed in the literature to account for such sex‐specific responsiveness. First, hormonal fluctuations across the menstrual cycle strongly modulate cortical excitability—estradiol has been shown to be associated with increased excitability, whereas progesterone has been shown to be associated with increased inhibition—leading to phase‐dependent variability in responses to brain stimulation (Inghilleri et al. 2004; Smith et al. 1999, 2002). Second, corresponding fluctuations in neurotransmitter levels, particularly GABA and glutamate, influence the excitation/inhibition balance that shapes tDCS effects (Epperson et al. 2002; Batra et al. 2008). Finally, structural differences in skull composition between women and men can alter current flow and the amount of stimulation reaching cortical tissue, potentially modulating stimulation efficacy (Russell et al. 2014). While these factors are not exhaustive, they illustrate a meaningful set of neurobiological variables that may contribute to sex differences in tDCS outcomes.

Because (1) our study is the first to report a moderation of HD‐tDCS effects on effort intensity, and (2) we did not directly assess these biological variables, further research is required to validate this finding and clarify the underlying mechanisms. This need is reinforced by the fact that our sample included more women than men and consisted exclusively of university students, which may limit generalizability. Accordingly, the present results should be considered as preliminary evidence that frontal hemispheric asymmetries induced through HD‐tDCS influence effort intensity for reward, particularly in women.

### 4.3 Prior Evidence on Approach–Avoidance Motivation

Our findings for women align with earlier research on the effects of neuromodulation on approach and avoidance motivation (Fecteau et al. 2007; Knoch, Gianotti, et al. 2006; Knoch, Pascual‐Leone, et al. 2006; Ohmann et al. 2018). In one study, researchers applied inhibitory TMS to the left or right dlPFC during a risk‐taking task. They found that participants who received TMS in the right dlPFC tended to choose higher rewards with lower probabilities than safer, smaller rewards. In this task, the participants completed 100 trials in which they were shown six horizontal boxes (a mix of blue and pink boxes), with the ratio of blue to pink boxes varying across the trials, and were asked to select the color of the box they believed contained a token and receive rewards for correct choices and penalties for incorrect choices. Riskier decisions, such as selecting a pink box with only a 1/6 chance of containing a token, were associated with higher rewards. The results demonstrated that participants made significantly riskier decisions following disruption in the right dlPFC (resulting in increased relative activity in the left frontal area) but not in the left dlPFC (resulting in increased relative right frontal activity) (Knoch, Gianotti, et al. 2006).

Most importantly, Ohmann et al. (2018) explored the effects of left anodal stimulation (LAS) in a decision‐making task designed to assess participants' reward motivation, specifically, their willingness to increase effort to gain a reward. In this task, participants were presented with two types of trials: an “easy” trial with a lower reward and a “difficult” trial with a higher reward and had to choose between completing the easy trial, which required less effort for a smaller reward, and the difficult trial, which required more effort for a larger reward. Participants received either left anodal or sham stimulation. The findings indicated that LAS increased the likelihood of participants selecting a more difficult trial with a higher reward. This outcome aligns with the notion that LAS enhances relative left FHA relative to the right, which is associated with approach motivation and increased SR.

### 4.4 Cardiovascular Effects

At the physiological level, our Bayesian statistical analyses found that women showed increased effort after RCS compared to LCS and sham stimulations, as indicated by PEP and SBP reactivity. However, our brain stimulation manipulation did not yield conclusive results for HR and DBP responses, although the reactivity patterns were generally consistent with those observed for PEP and SBP. This aligns with the fact that PEP is the most sensitive measure of beta‐adrenergic sympathetic impact on the heart and thus effort intensity (Gendolla 2025a; Kelsey 2012; Richter et al. 2008; Wright 1996). The observed corresponding effect on SBP reactivity is not surprising, as SBP is strongly influenced by cardiac output, which is related to cardiac contractile force. Also, previous studies have documented corresponding effects on both PEP and SBP responses (e.g., Brinkmann et al. 2009; Falk et al. 2022a; Framorando and Gendolla 2019a; Framorando et al. 2024a; Gendolla and Silvestrini 2011; Mazeres et al. 2019; Richter et al. 2008; Richter and Gendolla 2009; Silvestrini and Gendolla 2011). Importantly, in our present study, PEP reactivity was not accompanied by simultaneous decreases in blood pressure or HR. Accordingly, the observed PEP responses can be attributed to beta‐adrenergic sympathetic nervous system impact rather than cardiac preload or vascular afterload effects (see Sherwood et al. 1990).

### 4.5 Performance Effects

In the current experiment, no effects were observed on reaction times or response accuracy in the short‐term memory task. Previous research on effort has produced mixed results: While some studies have identified corresponding effects on effort‐related cardiovascular response and task performance (Framorando and Gendolla 2018; Gendolla and Silvestrini 2010), others have not observed such effects (Framorando and Gendolla 2019a, 2019b; Framorando et al. 2021; Lasauskaite Schüpbach et al. 2014; Wang et al. 2023). These inconsistencies may stem from the complex relationship between effort and performance, which makes it challenging to predict their connections. Effort intensity (behavioral input) and performance (behavioral output) are conceptually distinct, and performance depends not only on effort but also on factors such as task‐related capacity, persistence, and strategies (Locke and Latham 1990). For example, some may prioritize speed over accuracy, whereas others may focus more on accuracy at the expense of speed. Consequently, changes in effort intensity may not be directly reflected in performance outcomes. Finally, our mood measures did not provide any evidence that the tDCS induced changes in feeling states. This lack of evidence for stimulation effect on conscious affect contradicts the possibility that tDCS could have impacted effort through its effect on participants' mood (see Gendolla 2025b; Gendolla and Brinkmann 2005).

### 4.6 dlPFC Stimulation and Autonomic Activity

Recent work suggests that tDCS applied over the dlPFC may elicit automatic autonomic nervous system responses unrelated to motivational mechanisms (Carnevali et al. 2020; Sesa‐Ashton et al. 2022). To evaluate this possibility, we conducted a separate experiment (Framorando et al. 2025) in which we compared the effects of left versus right dlPFC stimulation across two task contexts: a fixed‐and‐easy task, where participants were informed that 50% accuracy would secure CHF 7.5 (approximately EUR 8.5), and an unfixed difficulty task, where each response yielded between CHF 0 and CHF 0.30 (approximately EUR 0.32) depending on speed and accuracy. This design allowed us to disentangle a potential global tDCS‐induced autonomic activation account from the effort predictions derived from MIT (Brehm and Self 1989). According to MIT, effort should increase with success importance only when task demand is unfixed or unclear, whereas a fixed‐and‐easy task should elicit uniformly low effort (e.g., Bouzidi et al. 2022). This implies that stimulation effects—if they modulate success importance—should emerge only in the unfixed task. The results confirmed this prediction: RCS compared to LCS increased effort only in the unfixed task, whereas both stimulation conditions produced similarly low effort in the fixed‐and‐easy context. Such a selective pattern is difficult to reconcile with the idea of automatic autonomic changes following tDCS, which should have affected effort in both task contexts. Instead, the findings converge on the interpretation that RCS increased relative left frontal activity, thereby increasing approach motivation and SR, which in turn determine higher effort when task demand is unfixed.

### 4.7 Cathodal Stimulation and Frontal Hemispheric Asymmetry

In the current study, we used cathodal stimulation to induce changes in frontal hemispheric asymmetry. This choice reflects our focus on functional connectivity, specifically the balance between the left and right dlPFC, rather than isolated regional excitability. Past research has shown that cathodal tDCS can modulate prefrontal connectivity, especially resting‐state network connectivity (Bertocci et al. 2021; Sehatpour et al. 2021). However, some overviews have emphasized that cathodal effects are less consistent across cognitive domains than those of anodal stimulation, highlighting substantial variability across studies (e.g., Jacobson et al. 2012; Ostrowski et al. 2022). Therefore, Jacobson et al. (2012) proposed several explanations for the variability in cathodal effects. First, the inhibition of one node may not matter if other regions in the network can compensate. Second, and similar to the first argument, many cognitive functions (e.g., language and executive control) recruit both hemispheres. If cathodal stimulation reduces excitability in one hemisphere, the other side could take over, limiting observable behavioral effects. Third, the impact of cathodal stimulation depends on the initial activation state of the targeted region. During cognitive task engagement, this could reduce the capacity of cathodal stimulation to further suppress neural firing and produce measurable inhibition.

Importantly, all these challenges are addressed in the current study's design. Concerns about compensation within the network or by the opposite hemisphere are less relevant here, as effects related to relative FHA do not rely on the functioning of a single region. In addition, since our theoretical framework focuses on relative hemisphere activation, absolute baseline activity in each dlPFC is not important. What matters in the present research is the balance between the two hemispheres rather than their respective absolute activity levels.

Taken together, these considerations provide a strong rationale for the use of cathodal HD‐tDCS in the context of relative FHA. However, future research is needed to determine whether comparable effects can also be obtained with anodal stimulation, and it would be particularly informative to combine cathodal protocols with concurrent EEG as mentioned below in order to directly track changes in functional connectivity.

### 4.8 Limitations

One limitation of the present study concerns the absence of EEG assessment of frontal hemispheric asymmetry. Our interpretation of the effects on PEP builds on previous findings showing that cathodal stimulation decreases the excitability of the targeted cortical area and on the widely assumed neuromodulatory effect that reducing activity in one hemisphere increases activity in the contralateral hemisphere (e.g., Fecteau et al. 2007; Smirni et al. 2015; see Schutter et al. 2004, for a review). Accordingly, we assume that RCS decreased activity in the right hemisphere and thereby increased activity in the left hemisphere, resulting in a biological state of approach motivation (see Kelley et al. 2017). Approach motivation should, in turn, increase reward sensitivity and the importance of success in a task with monetary incentives, which—under conditions of unclear task difficulty—determines results in higher effort. Although the consistency of our findings with established theoretical frameworks clearly supports the validity of this interpretation, it remains indirect. Future research combining HD‐tDCS with EEG is needed to improve the understanding of the neural mechanisms triggered by brain stimulation that underlie the observed cardiovascular responses.

### 4.9 Conclusion

In summary, our results contribute to the research on approach motivation and effort by showing that a manipulation of frontal hemispheric asymmetries using HD‐tDCS impacts effort‐related cardiovascular responses during a mental concentration task with a moderate monetary incentive. Notably, the expected effect was observed only in women, whereas men exhibited contrasting neuromodulatory effects. As this is the first study to identify sex differences in the effect of tDCS on effort intensity, further research is needed to validate these findings and elucidate the underlying mechanisms, particularly given the larger number of women than men in the sample. Overall, our findings provide initial evidence that manipulation of approach motivation through HD‐tDCS‐induced changes in frontal hemispheric asymmetry can influence mental effort differently based on sex.

## Author Contributions

**David Framorando:** conceptualization, investigation, funding acquisition, writing – original draft, methodology, writing – review and editing, project administration, data curation, supervision, formal analysis, software. **Guido H. E. Gendolla:** methodology, writing – review and editing. **Philip A. Gable:** methodology, writing – review and editing.

## Conflicts of Interest

The authors declare no conflicts of interest.

## Supporting information


**Table S1:** Cell means and standard errors (in parentheses) of cardiovascular baseline scores.
**Table S2:** Cell means and standard errors (in parentheses) of cardiovascular reactivity scores.

## Data Availability

The data and data coding for the reported studies are available on Yareta, the open‐access data archiving server at the University of Geneva: https://doi.org/10.26037/yareta:xrpzpx6psng3znp5f6e6lpem7e.

## Endnotes

^1^
In our AsPredicted preregistration, we indicated that extreme values would be winsorized at ±3 SD. However, in the actual analyses, we excluded participants whose values exceeded this threshold. This discrepancy stems from a mistake in the preregistration. The intended approach—consistent with the lead author's established practice—was participant‐level exclusion (e.g., Framorando et al. 2023; Framorando et al. 2024a, 2024b). For all analyses, we therefore excluded participants with excessive high or low accuracy (*n* = 2), HR (*n* = 2), and SBP (*n* = 1; already excluded due to excessive HR). No participant was excluded due to excessive low or PEP or DBP . The last excluded participant was because of physiological data loss. To reproduce the analysis samples, filter cases with Include_PEP == 0, Include_SBP == 0, Include_DBP == 0, and Include_HR == 0.

^2^
After data exclusion, the distributions of women and men were still balanced across the conditions: right cathodal (20 women and 13 men), left cathodal (21 women and 11 men), and sham (20 women and 12 men).

^3^
None of the enrolled participants reported having a pacemaker or other medical implants, or taking psychoactive drugs.

^4^
Besides “female” and “male,” participants were given a third option labeled “other” to indicate non‐binary gender identification. No participant selected this option.

^5^
Our preregistration on AsPredicted was intended to specify the main hypothesis‐driven analyses. Standard preliminary analyses commonly used in our prior work on effort (e.g., Framorando et al. 2023; Framorando and Gendolla 2023; Framorando et al. 2024a, 2024b) were not included in the preregistration but are reported here for consistency with our previous work and to provide a complete picture of the data. The preregistration primarily concerned the analysis of PEP reactivity, our main index of effort. All other analyses were not preregistered.

^6^
The specific ‐2 (right cathodal) +1 (left cathodal) +1 (sham) contrast was preregistered for the PEP reactivity analysis. However, preliminary analyses (non‐preregistered) indicated an interaction involving sex, which led us to conduct this contrast post hoc (non‐preregistered) separately for male and female participants.

^7^
For men, based on mean inspection, we post hoc exploratory tested a model with opposite weight contrasts (+2−1−1) using Bayesian factors. The test provided strong evidence in favor of the model for men (BF = 30.008, *P(M|data)* = 0.969). Mean after right cathodal stimulation was lower (M = −3.38, SE = 1.49) compared to left cathodal (M = −9.84, SE = 2.12) and sham stimulation conditions (M = −7.36, SE = 2.07).

^8^
For men, based on mean inspection, we post hoc exploratory tested a model with opposite weight contrasts (−2+1+1) using Bayesian factors. The test provided moderate evidence in favor of the model for men (BF = 8.329, *P(M|data)* = 0.895). Mean after right cathodal stimulation was lower (M = 7.35, SE = 1.32) compared to left cathodal (M = 11.42, SE = 1.58) and sham stimulation conditions (M = 8.51, SE = 1.45).
