# Supplementary material for: Approach Motivation and Reward Sensitivity: Effects of High‐Definition Transcranial Direct Current Stimulation (HD‐tDCS) to Brain Hemispheres on Effort‐Related Cardiovascular Response
Source: Eur J Neurosci. 2026 Feb 2;63(3):e70404. doi: 10.1111/ejn.70404 (PMC12865145; doi:10.1111/ejn.70404)
Supplement: Supplementary file 1 — Table S1: Cell means and standard errors (in parentheses) of cardiovascular baseline scores. Table S2: Cell means and standard errors (in parentheses) of cardiovascular reactivity scores. [file EJN-63-0-s001.docx]

**Supplemental Material**

**Approach Motivation and Reward Sensitivity: Effects of HD-tDCS Brain Hemisphere Stimulation on Effort-Related Cardiovascular Responses**

David Framorando^12^, Guido H. E. Gendolla^12^, Philip A. Gable^3^

^1^ Geneva Motivation Lab, FPSE, Section of Psychology, University of Geneva, Geneva, Switzerland

^2^ Swiss Center for Affective Sciences, University of Geneva, Geneva, Switzerland

^3^ Department of Psychology, University of Delaware, Newark, DE, USA

Cardiac output (CO) and total peripheral resistance (TPR) were also analyzed to provide interested readers with a more comprehensive picture of the hemodynamic response during task performance, although neither measure was relevant for our hypotheses. One participant was excluded from the analysis because of excessive CO and TPR reactivity (> 3SDs than condition Ms) and two participants were excluded because of excessively low response accuracy (> 3 *SD*s from the condition mean). CO was calculated by the Cardioscreen system according to the Sramek and Bernstein formula (see Bernstein, 1986). TPR was derived from CO and mean arterial pressure (MAP = 2 x DBP + SBP / 3) according to the formula TPR = (MAP / CO) * 80 (Sherwood et al., 1990). Given the absence of specific hypotheses for the two hemodynamic indices, both CO and TPR were analyzed with 3 (stimulation condition) x 2 (sex) Bayesian ANOVAs.

**CO Baseline Values**

CO values were constituted by averaging the cardiovascular values of the last three minutes of the habituation phase (Cronbach's αs > .99). The mean values and standard errors of the cells are presented in Supplemental Table S1. The Preliminary Bayesian ANOVA indicated that the best-fitting model for CO cardiovascular baseline scores included only the gender factor (*P(M|data)* = 0.437) (Men: *M* = 5.71; *SE* = 0.14; Women: *M* = 5.37; *SE* = 0.10). Models including the stimulation condition, and stimulation condition x sex interaction revealed lower posterior distribution fits (*BF* < 0.350; *P(M|data)* < 0.117). These findings do not suggest evidence for condition-based differences at baseline.

**TPR Baseline Values**

TPR values were constituted by averaging the cardiovascular values of the last three minutes of the habituation phase (Cronbach's αs > .97). The mean values and standard errors of the cells are presented in Supplemental Table S1. Preliminary Bayesian ANOVA of TPR cardiovascular baseline scores showed that the results mostly supported the null effect model (*P(M|data)* = 0.691). Models including, stimulation condition, sex, and stimulation condition x sex interaction effects revealed lower posterior distribution fits (*BFs* < 0.278, *P(M|data)* < 0.192). These findings do not suggest evidence for condition-based differences at baseline.

| **Supplemental Table 1**  Cell means and standard errors (in parentheses) of cardiovascular baseline scores. | | | | | | |
| --- | --- | --- | --- | --- | --- | --- |
|  | Men | | | Women | | |
|  | Right cathodal | Left cathodal | Sham | Right cathodal | Left cathodal | Sham |
| CO | 5.93 (0.29) | 5.80 (0.21) | 5.42 (0.21) | 5.26 (0.17) | 5.56 (0.20) | 5.27 (0.14) |
| TPR | 1027.36 (51.88) | 1064.38 (56.26) | 1093.50 (46.89) | 1141.69 (40.52) | 1058.57 (48.60) | 1068.05 (31.70) |
| *Note*: CO = cardiac output (in liters per minute), TPR = total peripheral resistance (in dynes second per centimeter to the 5th power). *N* = 99 for both measures. | | | | | | |

**CO and TPR Reactivity**

Reactivity scores for the CO and TPR indices were created by subtracting the baseline values from the five 1-min values assessed during the task and averaging these values (Cronbach's αs > .90). Means and standard errors are shown in Supplemental Table S2.
 ***CO and TPR Reactivity.*** Bayesian ANOVAs of CO and TPR reactivity showed that the results mostly supported the null effect models (CO reactivity: *P(M|data)* = 0.532; TPR reactivity: *P(M|data)* = 0.497). Models including, stimulation condition, sex, and stimulation condition x sex interaction effects revealed lower posterior distribution fits (CO reactivity: *BFs* < 0.509, *P(M|data)* < 0.271; TPR reactivity: *BFs* < 0.752, *P(M|data)* < 0.374). This does not provide evidence that the stimulation condition, sex, or their interaction affected CO or TPR reactivity.

| **Supplemental Table 2**  Cell means and standard errors (in parentheses) of cardiovascular reactivity scores. | | | | | | |
| --- | --- | --- | --- | --- | --- | --- |
|  | Men | | | Women | | |
|  | Right cathodal | Left cathodal | Sham | Right cathodal | Left cathodal | Sham |
| CO | 0.23 (0.10) | 0.64 (0.15) | 0.48 (0.15) | 0.33 (0.08) | 0.32 (0.08) | 0.31 (0.07) |
| TPR | 44.94 (19.75) | -15.38 (35.41) | 21.11 (24.58) | -13.14 (14.62) | -3.25 (17.47) | -7.00 (12.95) |
| *Note*: CO = cardiac output (in liters per minute), TPR = total peripheral resistance (in dynes second per centimeter to the 5th power). *N* = 99 for both measures. | | | | | | |

**Supplemental References**

Bernstein, D. P. (1986). A new stroke volume equation for thoracic electrical
 bioimpedance: Theory and rationale. *Critical Care Medicine, 14*, 904-909.
 <https://doi.org/>10.1097/00003246-198610000-00017

Sherwood, A., Allen, M. T., Fahrenberg, J., Kelsey, R. M., Lovallo, W. R., & Doornen, L. J. P. (1990). Methodological guidelines for impedance cardiography. *Psychophysiology*, 7, 1–23. <https://doi.org/10.1111/j.1469-8986.1990.tb02171.x>
